# Pubertal Maturation and Trajectories of Depression During Early Adolescence

**DOI:** 10.3389/fpsyg.2019.01362

**Published:** 2019-06-12

**Authors:** Taylor C. McGuire, Kathleen C. McCormick, Mary Kate Koch, Jane Mendle

**Affiliations:** ^1^ Department of Psychiatry, University of Michigan, Ann Arbor, MI, United States; ^2^ Department of Psychology, University of Illinois Urbana-Champaign, Champaign, IL, United States; ^3^ Department of Human Development, Cornell University, Ithaca, NY, United States

**Keywords:** adolescent, female, menarche, puberty, depression, rejection, peers

## Abstract

Beginning at puberty, prevalence of depression in females rises dramatically. The physical changes of puberty coincide with a period of social flux, during which relationships become less stable and more prone to conflict. While this social upheaval is normatively distressing for girls, it may be especially so for girls with cognitive styles that leave them more susceptible to depression. The present study investigated depressive symptoms at two time points during early pubertal maturation. *N* = 110 girls (*M*_age_ = 11.57, SD = 0.98) reported on depressive symptomology, pubertal maturation, ruminative coping style, frequency of peer conflict, and rejection sensitivity. Multivariate analyses suggest more advanced pubertal development and greater rejection sensitivity at Time 1 predicted higher levels of depressive symptoms at Time 2, after accounting for baseline levels of depressive symptoms and all other social and cognitive correlates of depression. This effect was also found in early maturing girls. Menarche status was not significant. Since menarche occurs toward the end of puberty, results suggest that risk for worsening depression is not associated with completing puberty, or with menstruation itself. Rather, increases in depressive symptoms seem to be associated with physical changes that emerge early in the pubertal transition, especially for early maturing girls, paired with anticipatory concerns about social rejection.

## Introduction

The major reproductive milestones in women’s lives – puberty, menstruation, childbirth, and menopause – all encompass major biological, psychological, and socioemotional changes. Puberty, the first reproductive milestone, refers to the physical growth and sexual maturation that indicates passage from childhood into adolescence. Because puberty occurs earlier in the life span, the complex changes and experiences during this period have the potential to alter developmental trajectories. Changes at puberty are especially notable because of the concurrent shift in depression vulnerability. Findings from epidemiological research suggest that rates of depression are approximately equal between sexes before the onset of puberty, after which, rates for depression in girls are approximately twice that of boys (Bromet et al., 2011; Salk et al., 2017). While puberty alone may play an integral role in the increased risk of depression, the psychological changes associated with adolescence are also intimately intertwined with contemporaneous biological and sexual maturation. The present study investigates how biopsychosocial changes during puberty contribute to and predict exacerbations of depressive symptoms in girls during the early adolescent window.

There are a variety of changes girls must adjust to during puberty. The onset of puberty is marked by significant rises in adrenal and gonadal hormones and the development of primary and secondary sex characteristics. It takes, on average, 4–5 years to progress through puberty (Finer and Philbin, 2013). *Pubertal status* is conceptualized as the level of development through visible bodily shifts in breasts, body shape, height, skin, and body hair. More advanced pubertal status, or physical development, has been linked to depressive disorders and is thought to help explain gender differences in rates of depression (Hankin and Abramson, 2001; Conley and Rudolph, 2009; Lewis et al., 2018). *Pubertal timing*, or the relative status of one’s development compared to same-sex peers, has also been shown to have significant associations with the development of internalizing symptoms (Ullsperger and Nikolas, 2017). Prior research suggests that early maturing girls tend to exhibit more severe levels of depressive symptoms than girls who mature at the same time as or later than most peers (Negriff and Susman, 2011; Graber, 2013; Lewis et al., 2018; Mendle et al., 2018; Hamlat et al., 2019).

In addition to tracking the development and timing of visible indicators of pubertal maturation (e.g., skin changes, hair growth, etc.), it may be important to consider menarche. Menarche, or the start of menstruation, is one of the final pubertal milestones and represents the completion or near-completion of sexual maturation. Some research suggests age at menarche shows different patterns of association with symptoms of depression and other psychopathologies compared to other pubertal indicators like breast development (Harden et al., 2012; Carter et al., 2013; Kretsch et al., 2016). These findings demonstrate the need to question whether entering, enduring, or completing puberty poses a greater risk for depression.

The onset of puberty typically marks the entry into adolescence, or the developmental period that connects childhood and adulthood. Early adolescence, or the period from the onset of puberty to 14 or 15 years of age (Sawyer et al., 2012), represents a distinctly turbulent period of socioemotional change. The shifting social landscape during this time places greater importance on peer relationships and social status. With this increased focus on social relationships, girls are also susceptible to experiencing more negative social stressors that may contribute to vulnerability for depression. As girls renegotiate friendships and relationships, interpersonal conflicts and fears of social rejection can become more prominent. Conflict with peers contributes to internalizing symptoms in adolescent girls (Purdie and Downey, 2000). In addition, rejection sensitivity, or the tendency to expect and readily perceive rejection from others, has been associated with increased psychopathology in adolescence and adulthood (Purdie and Downey, 2000; Ayduk et al., 2001; Marston et al., 2010; Zhou et al., in press). Individuals who experience higher levels of rejection sensitivity are more likely to perceive rejection in the face of ambiguous stimuli and are more sensitive to both the potential for and likelihood of being rejected by others. Further, girls with high rejection sensitivity are particularly likely to experience peer conflict and relationship difficulties (Purdie and Downey, 2000). Research suggests that being liked by peers predicts a reduction of rejection sensitivity in adolescents (London et al., 2007). Consequently, higher levels of rejection sensitivity and peer conflict during puberty could color perceptions of peer relationships as particularly fraught and uncertain, which in turn could generate more interpersonal stress and vulnerability for depression.

These myriad biological and socioemotional changes may be particularly distressing to girls utilizing maladaptive coping strategies. Rumination is an emotion regulation strategy that may be used to cope with stressors by perseverating on the causes, consequences, and factors involving one’s distress. Because rumination is linked to negative affect, it has been studied as a precursor to depression in childhood, adolescence, and adulthood (Nolen-Hoeksema et al., 1993; Gibb et al., 2012; Gomez-Baya et al., 2017), and has been implicated in the rise in depressive symptoms for girls at puberty (Mezulis et al., 2014; Alloy et al., 2016). In addition, there is evidence to suggest that adolescent girls are more likely to ruminate than preadolescent girls (Hampel and Petermann, 2005) and that early adolescents who ruminate tend to report more difficulties in their peer relationships and greater symptoms of internalizing (McLaughlin and Nolen-Hoeksema, 2012). Given the socially tumultuous nature of adolescence, girls who experience more peer conflict and are higher in rejection sensitivity may be made further vulnerable by a cognitive style that has been found to increase negative affect (Nolen-Hoeksema et al., 1993).

### The Present Study

The present study investigated trajectories of depressive symptoms specifically during early reproductive maturation. Because puberty is a time when interpersonal interactions become increasingly salient and fraught for girls, we sought to examine the contributions of common biopsychosocial correlates of depression – rumination, rejection sensitivity, peer conflict, and level of pubertal development – with exacerbations in depressive symptomology over a 4-month period. We also examined these biopsychosocial correlates with respect to pubertal timing, considering whether these symptom trajectories might be intensified in girls who matured earlier than peers. While previous research has identified the interplay of pubertal and psychosocial changes in relation to psychopathology (see review articles; Musliner et al., 2016; Shore et al., 2018), very few studies, to date, have explored the constellation of variables in the present study and clarified mechanisms of risk and vulnerability during the early adolescent transition. We therefore targeted two specific research questions. First, are girls with more advanced pubertal status more likely to experience depressive symptoms at follow-up, after accounting for baseline levels of depression, rumination, rejection sensitivity, and interpersonal difficulties? Second, does this pattern of findings hold for early maturing girls?

## Materials and Methods

### Participants

Participants comprised *N* = 110 girls (*M_age_* = 11.57, range = 9–14) recruited from a research partnership established with (New York State 4-H) youth summer programs between 2015 and 2017. All girls in the target age range were invited to participate Girls were administered a questionnaire battery at the time of recruitment and a 4-month follow-up survey. For the purposes of this study, only participants who completed the follow-up survey were included in analyses. In this sample, youth self-identified as primarily European American (80%), Black or African American (2.7%), American Indian or Native American (3.6%), East Asian/Pacific Islander (3.6%), Southeast Asian (0.9%), or another racial/ethnic background (7.3%). Two percent of girls self-identified as Hispanic/Latina. The study was approved by the Institutional Review Board at Cornell University, IRB Protocol #1207003173. Parental/guardian written informed consent and youth written assent were obtained from participants.

### Measures

#### Reproductive Maturation

The Pubertal Development Scale (PDS; Petersen et al., 1988) was used to assess pubertal status, pubertal timing, and menarcheal status. Pubertal status was operationalized using four items assessing changes in height, body hair, skin (i.e., acne), and breast growth. Items were measured using a four-point Likert scale from 1 (= *has not yet begun*) to 4 (= *seems completed*) and summed for a composite score. The mean composite PDS score in this sample was 9.90 (SD = 2.65, range = 3–16), with higher scores indicating greater overall physical development. To operationalize pubertal timing, composite PDS scores were standardized according to year of chronological age, so that higher scores indicate greater levels of physical development relative to girls of the same age. Menarcheal status was assessed using one dichotomous indicator from the PDS: “Have you begun to menstruate (get your period)?”

#### Depression

The Center for Epidemiological Studies-Depression Scale for Children (CES-DC; Radloff, 1977) is a 20-item self-report questionnaire used to assess depressive symptomatology in children. Scores on the CES-DC range from 0 to 60, with higher scores indicating greater endorsement of depressive symptoms. A score of 16 is typically used to signify a clinically relevant level of symptoms. In this sample, the mean score at baseline was 15.72 (SD = 11.07, range = 0–56). The mean score at 3-month follow-up was 14.74 (SD = 11.53). Internal reliability was good at Time 1 (*α* = 0.90) and excellent at Time 2 (*α* = 0.93).

#### Peer Relations

The Index of Peer Relations Scale (IPR; Hudson, 1992; Forte and Green, 1994) was used to ascertain the severity of peer relationship problems and social adjustment difficulties. The IPR comprises 25 statements rated on a seven-point Likert scale, ranging from 1 (none of the time) to 7 (all of the time). The present study modified the wording of the IPR to substitute the phrase “kids my age” for “peers.” Sample items include: “Kids my age don’t seem to even notice me,” “I really feel left out of the group of kids my age,” and “I hate kids my age.” Scores on the IPR range from 0.67 to 100, with higher scores indicative of greater peer conflict. A score at or above 30 typically signifies a clinically relevant level of peer problems and a score at or above 70 indicates severe distress. In the current study, the mean score was 28.39 (SD = 16.46, range = 0–84.67). Internal reliability was excellent (*α* = 0.95).

#### Rejection Sensitivity

The Children’s Rejection Sensitivity Questionnaire 6-Item Form (CRSQ; Downey et al., 1998) was used to measure children’s propensity to respond defensively to social rejection or anticipated social rejection. Children are asked to determine the degree of anxiety and anger they would feel in six social rejection scenarios, with responses scored on a six-point Likert scale, from 1 (not nervous/mad) to 6 (very, very nervous/mad). The CRSQ also queried perceived likelihood of a positive outcome for each scenario, with responses scored on a six-point Likert scale, from 1 (YES!!!) to 6 (NO!!!). Anger and anxiety items were multiplied separately by each likelihood item, and all six item scores were averaged to create a total score. Higher scores are indicative of greater rejection sensitivity. The total rejection sensitivity mean score in the sample was 17.36 (SD = 8.97, range = 3–49). The anger sub-scale mean was 7.58 (SD = 4.39; range = 1.17–25.67) and the anxiety sub-scale mean was 9.78 (SD = 5.14; range = 1.83–25). Internal reliability was good (*α*_total_ = 0.85; *α*_anger_ = 0.79; *α*_anxiety_ = 0.80).

#### Ruminative Coping

Ruminative coping was assessed using the Ruminative Response Scale of the Children’s Response Styles Questionnaire (CRSQ-R; Abela et al., 2002). Sample items include: “When you’re sad, you think about how sad you feel” and “When you’re sad, you think about a recent situation wishing it had gone better.” Scores on the CRSQ-R ranged from 0 to 39, with greater scores indicating higher ruminative thinking. The mean score was 13.58 (SD = 9.01, range = 1–39). Internal reliability was good (*α* = 0.89).

### Analytic Plan

We investigated how biopsychosocial factors predicted depression at Time 2 using two three-stage hierarchical linear regressions. The pubertal status model predictor variables included rejection sensitivity, rumination, peer conflict, pubertal status (with age, scaled in months and years, as a covariate). The pubertal timing model included the same predictor variables; however, pubertal timing was included instead of pubertal status and age was not included as a covariate, as chronological age is taken into account in the operationalization of pubertal timing. Descriptive analyses and hierarchical linear regressions were conducted using SPSS 25 (IBM Corp, 2016). Continuous predictor variables were centered at their means prior to analysis. All variables met assumptions of linear regressions. Step 1 was a simple linear regression predicting depressive symptoms at Time 2 from depressive symptoms at Time 1. Depressive symptoms at Time 1 were entered at Step 1 to control for depressive symptoms at Time 2. At Step 2, cognitive and social risks for depression (peer conflict, rejection sensitivity, and rumination) were added. At Step 3, reproductive maturation variables (i.e., PDS scores and menarche status) were added to the model. The variables were structured to assess whether the pubertal variables predicted depression above and beyond the effect of the social and cognitive risk factors and baseline levels of depressive symptoms.

## Results

### Descriptive Analyses

Table 1 depicts descriptive statistics and bivariate Pearson correlations of all variables. The psychosocial variables (rumination, rejection sensitivity, and peer conflict) were significantly intercorrelated. Reproductive maturation variables were not significantly correlated with the psychosocial variables, except for a small correlation between pubertal status and rumination (*r* = 0.20). Time 2 depressive symptomatology was significantly correlated with all biopsychosocial predictor variables, ranging from menstruation and pubertal timing (*r* = 0.23) to Time 1 depressive symptomology (*r* = 0.58).

**Table 1:** Descriptive statistics.

	1	2	3	4	5	6	7	8
T1 depression	–							
Rumination	0.69^***^	–						
Peer conflict	0.46^**^	0.34^**^	–					
Rejection sensitivity	0.52^**^	0.46^**^	0.62^**^	–				
Pubertal Development Scale	0.16	0.20^*^	0.13	0.07	–			
Age	0.12	0.12	0.17	0.12	0.39^**^	–		
Menstruation	0.11	0.07	0.04	0.05	0.41^**^	0.44^**^	–	
T2 Depression	0.58^***^	0.47^**^	0.42^**^	0.51^**^	0.34^**^	0.34^**^	0.23^*^	–
Mean	15.73	13.58	28.40	17.36	9.90	11.57	1.87	14.74
SD	11.07	9.01	16.46	8.97	2.65	0.98	1.37	11.53

* p < 0.05;

** p < 0.01;

*** *p < 0.001*.

### Hierarchical Regressions

Full results of the hierarchical regression models are summarized in Tables 2 and 3. Girls reporting higher levels of depressive symptomology at Time 1 were more likely to report experiencing more depressive symptomology at follow-up (*β*_status_ = 0.36, *p* = 0.001; *β*_timing_ = 0.36, *p* = 0.001). Additionally, higher levels of rejection sensitivity at Time 1 significantly predicted higher levels of depressive symptoms at Time 2 (*β*_status_ = 0.29; *p* = 0.005; *β*_timing_ = 0.27; *p* = 0.008), even after accounting for Time 1 symptoms. Neither rumination (*β*_status_ = 0.02; *p* = 0.83; *β*_timing_ = 0.04; *p* = 0.73) nor peer conflict (*β*_status_ = 0.00; *p* = 0.99; *β*_timing_ = 0.04; *p* = 0.65) were significant predictors of depression in either the pubertal status or pubertal timing models. Girls with a more advanced pubertal status at Time 1 were more likely to report higher levels of depressive symptoms at Time 2 (*β*_status_ = 0.17; *p* = 0.04), after accounting for baseline levels of depressive symptoms and social and cognitive correlates of depression. Girls with early pubertal timing at Time 1 were also more likely to report higher levels of depressive symptoms at Time 2 (*β*_timing_ = 0.17; *p* = 0.03), after accounting for baseline levels of depressive symptoms and social and cognitive correlates of depression. Menarche status was not a significant predictor of depressive symptoms at Time 2 in either model (*β*_status_ = 0.03, *p* = 0.72; **β**_timing_ = 0.14, p = 0.07).

**Table 2:** Pubertal status regression estimates predicting Time 2 depressive symptomology.

Step	Variable	*β*	SE	*R* ^2^	Δ*R^2^*
Step 1	Depressive symptoms	0.36^**^	0.11	0.34	
Step 2	Peer conflict	0.00	0.07	0.41	0.07^***^
	Rumination	0.02	0.13		
	Rejection sensitivity	0.29^**^	0.13		
Step 3	Pubertal development	0.17^**^	0.36	0.50	0.09^***^
	Age	0.19^**^	0.98		
	Menarcheal status	0.03	2.12		

*β, standardized coefficient; SE, standard error; *R*^2^, percentage of variance accounted for at each step; Δ*R*^2^, change in *R*^2^ at each step*.

* p < 0.05;

** p < 0.01;

*** *p < 0.001*.

**Table 3:** Pubertal timing regression estimates predicting Time 2 depressive symptomology.

Step	Variable	*β*	SE	*R* ^2^	Δ*R* ^2^
Step 1	Depressive symptoms	0.36**	0.11	0.34	
Step 2	Peer conflict	0.04	0.07	0.40	0.07*
	Rumination	0.04	0.13		
	Rejection sensitivity	0.27**	0.13		
Step 3	Pubertal timing	0.17*	0.93	0.46	0.06**
	Menstrual status	0.14	1.95		

*β, standardized coefficient; SE, standard error; *R*^2^, percentage of variance accounted for at each step; Δ*R*^2^, change in *R*^2^ at each step*.

* p < 0.05;

** p < 0.01;

*** *p < 0.001*.

### Supplementary Analyses

Although our sample was relatively limited in racial and ethnic diversity (see discussion, below), a supplementary set of models for both pubertal status and pubertal timing were run with race/ethnicity included as a covariate. Race/ethnicity was coded in two ways: (1) all race/ethnicity options as separate dummy variables and (2) white and nonwhite dummy variables. Results did not change.

## Discussion

Depression in adolescent girls is both endemic and worrisome. The extant literature has examined psychosocial correlates and their associations with the trajectory of depressive symptoms (Mezulis et al., 2014; Musliner et al., 2016; Ellis et al., 2017; Shore et al., 2018). The present study extends prior findings by investigating how multiple indicators of pubertal maturation and psychosocial risk factors contribute to and predict exacerbations of depressive symptoms in early adolescent girls. Our results suggest an important coupling of pubertal maturation and rejection sensitivity. Girls with a more advanced pubertal status, earlier pubertal timing, and those who express a high level of concern about social rejection were more likely to report worsening depressive symptoms. Menarcheal status, peer conflict, and ruminative coping were not significant predictors of later depressive symptoms.

A strength of the present study is that it provides a foundational basis that begins to disentangle the complex role of puberty in concert with common psychosocial predictors of psychopathology. Our sample offered the opportunity to examine three indicators of pubertal development: pubertal timing, pubertal status, and menarcheal status. Consistent with our hypotheses, both pubertal status and pubertal timing played an independent role in depressive symptoms over and beyond the effect of baseline levels of depressive symptoms and psychosocial predictors: girls who were further along in the pubertal process and girls who were further along relative to girls of the same age were at a greater risk of reporting depressive symptomology. Completing menarche did not confer additional risk. Our results are a snapshot into early adolescence and underscore the need for further work to examine pubertal processes within a larger, longitudinal sample. Future studies can build upon the present study and identify how specific mechanisms of puberty uniquely contribute to exacerbated risk in girls.

Understanding why reproductive maturation poses psychological risk is a complicated task for researchers. Although hormonal changes are not singularly associated with increases in depression (Soares and Zitek, 2008), studying reproductive maturation can elucidate the relationship between hormones, heightened sensitivity to hormone changes, and vulnerabilities for the onset or worsening of depression. Prior research has shown that hormone changes throughout the menstrual cycle are associated with increases in stress reactivity, negative cognitive appraisals, and internalizing symptoms (Albert et al., 2015; Kiesner et al., 2016; Mulligan et al., 2018). Our study confirmed that even very early reproductive changes during puberty predict decrements in mood. Notably, menarche status did not predict depressive symptoms, which is not inconsistent with existing literature. Menarche, although a useful marker for development, does not indicate hormone levels or cyclical reproductive hormone change, and is only one indicator of pubertal development. The menstrual cycle can take between 3 and 4 years to stabilize post-menarche (Widholm and Kantero, 1971; De Sanctis et al., 2019). Therefore, the onset of menstruation itself is not a good indicator of an individual’s hormonal levels or the stability of their menstrual cycle and cyclical hormone change, which may explain why menarche status did not map onto depressive symptoms beyond pubertal status. Future research could explore how both the increase and variability of hormone levels during this period are associated with cognitive vulnerabilities to depression. Exploring the specific psychosocial changes that pubertal status signals can also inform our understanding of risk for depression exacerbation during the early pubertal transition. Additionally, studying the effects of hormonal changes occurring during adrenarche may further clarify the role that hormones have on depressive symptoms. A meta-analysis of adrenarche and mental health found an association between adrenarche and both internalizing and externalizing symptoms (Byrne et al., 2017); however, the authors also noted the deficit of research studying this period of early adolescent development.

Consistent with prior literature (Tops et al., 2008; Liu et al., 2014; Zhou et al., in press), our results also highlight the importance of adolescents’ anticipatory concerns about how others will perceive and respond to them in social settings. Recent work has examined the mechanism by which rejection sensitivity is related to depression, suggesting that stress generation may mediate the relationship between depression and rejection sensitivity (Liu et al., 2014). Specifically, individuals high in rejection sensitivity may behave in ways that elicit more conflict with peers, leading to greater interpersonal stress. Puberty represents a time of myriad new stressors, which could help explain how rejection sensitivity influences depression through increased relationship stress and sensitivity to rejection in these precarious peer relationships. Future work could examine how specific types of relationships could serve to ameliorate or worsen rejection sensitivity, stress, and depression.

Perhaps most notably, we did not find that either rumination or peer conflict were necessarily associated with worsening depression over time, after accounting for baseline levels of depressive symptoms. This result is surprising, given prior research that suggests rumination exacerbates depressive symptoms (Mezulis et al., 2014; Grierson et al., 2016) and mediates the effect of pubertal timing on depression (Alloy et al., 2016). One possibility is that rumination is not as robust a predictor of subsequent depression symptoms when more biopsychosocial indicators are simultaneously included in analysis. It is also possible that many of these variables are closely related and more complex modeling techniques, such as structural equation modeling, may be needed in order to tease apart relationships with depression. Finally, rejection sensitivity may serve as either a moderator or mediator between rumination and depression (Hilt et al., 2017). Findings from the adult literature suggest that rejection sensitivity is prospectively associated with rumination (Pearson et al., 2011), which suggests that individuals prone to perceiving rejection may be more likely to dwell on that rejection. Further research with additional time points may better clarify the relationship between these social-cognitive processes throughout adolescence.

Our study offers new insights into the early adolescent period, but – like all research – it is not without its limitations. These include the comparatively small sample size and short longitudinal follow-up time. In addition, while our sample demographics are consistent with the demographics of the region in which the data were collected, the sample is also limited by a lack of racial/ethnic diversity and cannot contribute to a broader understanding of how becoming reproductively mature may differ meaningfully for girls depending on their background. Research on pubertal development can be furthered by a focus on racially and ethnically underrepresented groups (Mendle et al., 2019), which have been historically understudied. Additionally, future studies should seek to better understand the experience of sexual minorities and how sexual and gender identity may be related to mental health during the pubertal transition. Finally, our analyses focus on girls, but it could be argued that a comparison across sexes might provide the best understanding of the unique vulnerabilities associated with female reproductive maturation.

Given these limitations, we view our study as a preliminary investigation into this topic, and hope that it serves as a foundation for future research. Analyzing depressive symptoms, rejection sensitivity, rumination, and peer conflict in tandem with reproductive maturation across two time points allows for a clearer picture of the potential causes and consequences of psychosocial factors commonly found to underlie depression. A follow-up investigation of these factors is an important future direction of this work in disentangling how puberty is a period of increased risk for worsening depressive symptoms in girls.

## Ethics Statement

This study was carried out in accordance with the recommendations of the Cornell University Institutional Review Board with written informed consent from all subjects. All subjects gave written informed consent in accordance with the Declaration of Helsinki. The protocol was approved by the Cornell University Institutional Review Board.

## Author Contributions

JM conceived and designed the experiment. TM, KM, and MK contributed to data collection. TM analyzed the data. TM and KM wrote the paper. JM, TM, KM, and MK provided feedback and revision of the text.

### Conflict of Interest Statement

The authors declare that the research was conducted in the absence of any commercial or financial relationships that could be construed as a potential conflict of interest.
